# Effects of foliar application of amino acid liquid fertilizers, with or without *Bacillus amyloliquefaciens* SQR9, on cowpea yield and leaf microbiota

**DOI:** 10.1371/journal.pone.0222048

**Published:** 2019-09-04

**Authors:** Dongsheng Wang, Xuhui Deng, Bei Wang, Na Zhang, Chengzhi Zhu, Zixuan Jiao, Rong Li, Qirong Shen

**Affiliations:** 1 Jiangsu Provincial Key Lab of Solid Organic Waste Utilization, Jiangsu Collaborative Innovation Center of Solid Organic Wastes, Educational Ministry Engineering Center of Resource-Saving Fertilizers, Nanjing Agricultural University, Nanjing, Jiangsu, China; 2 Nanjing Institute of Vegetable Science, Nanjing, Jiangsu, China; Estacion Experimental del Zaidin, SPAIN

## Abstract

Leaf surface fertilization with liquid fertilizer produced from amino acids constitutes a potentially important source of nitrogen and is important for plant production. However, few reports have focused on the plant growth promotion by novel liquid fertilizers created by new amino acid resources, let alone the influence on leaf microbiota. In this study, the effects of liquid fertilizer, created by amino acids hydrolyzed from animal hairs with or without the PGPR strain *Bacillus amyloliquefaciens* SQR9, on crop yield and leaf microbiota were investigated. The results showed that leaves sprayed with amino acid liquid fertilizer (AA) and liquid biological fertilizer (AA9) persistently increased cowpea yields compared to the control amended with chemical fertilizer (CF). Fertilization with amino acid fertilizer showed no significant difference in microbial composition compared with the CF treatment; however, the introduction of functional microbes altered the microbial composition. Pearson correlation analysis, VPA analysis and SEM models all revealed that the amino acids liquid fertilizer application, but not the functional strain or the altered microbiota, performed as the direct driver attributing to yield enhancement. We conclude that leaf fertilization with a novel amino acid liquid fertilizer can greatly enhance the crop yield and that the addition of beneficial microbes may perform the role in further altering the composition of leaf microbiota.

## Introduction

Agricultural intensification stimulates increased production of staple crops and leads to greater food security for a continuously growing world population [1, 2]. Intensive practices in modern agriculture through the extensive use of chemical fertilizers in soil also alter biotic interactions and influence patterns of resource availability in ecosystems [3], leading to increased awareness of adverse environmental impacts [4]. Thus, developing new types of fertilizer and exploring novel application patterns to ensure high fertilizer-use efficiency have caused wide concern among researchers, administrators of agriculture, and farmers. Nutrient uptake is performed primarily by plant roots [5], but nutrients can also be absorbed by leaves through foliar applications at adequate levels [6]. Since the early 1980s, a surge of studies have focused on foliar fertilizer application [7], which can induce fast absorption, high nutrient availability and high economic benefits; therefore, foliar fertilization is now becoming increasingly popular [8].

Currently, much attention has been devoted to the evaluation of the importance of dissolved organic nitrogen, particularly free amino acids and peptides, for plant uptake [9]. The importance of amino acids is attributed to their wide utilization for the biosynthesis of a large variety of different organic compounds [10]. Amino acids have already showed the greatest importance in plant nutrition for obtaining of higher yields and quality and shortening of the productive cycle with better dry material [11]. Considerable differences have been reported among fertilizer sources in burning foliage with foliar application of inorganic fertilizers, especially N [12]; however, there are few studies focusing on the plant growth promotion by foliar application of amino acids. Thus, liquid fertilizer produced by amino acids constitutes a potentially important source of nitrogen [13], and foliar application of the novel liquid fertilizer is important for plants in various ecosystems.

Plant growth-promoting rhizobacteria (PGPR) that exert beneficial effects on plant development have been widely used in soil to promote plant growth and suppress soil-borne disease [14, 15]. Often, beneficial microbes are employed as a root inoculant [16], while the foliar application and its subsequent effects are of less concern [17]. However, foliar application can avoid the adverse influences of many biotic and abiotic factors on the soil environment [18] and the plants can be treated throughout the whole season with the microbial inoculants, at certain growth stages to promote plant growth and improve plant resilience or at distinct weather conditions to fight plant diseases pests [19] due to PGPR can promote the plant growth by a wide variety of mechanisms such as phosphate solubilization, phytohormone production, induction of systemic resistance, and suppress pathogens [20]. PGPR sprayed on plant leaves not only have biocontrol function [21], but also could promote plant growth [22, 23]. Moreover, compound liquid amino acids have been already reported to enhance the PGPR activity [24]. Thus, the hypothesis of this study is that foliar application of PGPR, particularly combined with amino acids, will provide further beneficial to plant growth and offer a novel strategy for enhancing crop yield.

Plants in nature are colonized by a large, diverse array of nonpathogenic microbes [25], which are usually defined as phyllospheric and endophytic microbes that are assumed to play a key role in the metabolism of host plants [26]. The global population of phyllosphere bacterial population is estimated to be ~10^26^ cells [27], and cell densities in the phyllosphere are typically approximately 10^6^ to 10^7^ cells cm^-2^ [25]. Recently, studies have been performed to examine the relationship between foliar fertilization, which has recently become popular in plant production, and plant yield [6, 7]. However, how the foliar application of amino acids, let alone combining amino acids with PGPR, alters leaf microbiota has attracted less attention and remains unclear.

In this study, amino acids hydrolyzed from animal hairs and a PGPR strain, *Bacillus amyloliquefaciens* SQR9, with effective plant growth promotion and various pathogen suppression abilities [28] were selected to create a liquid fertilizer (only amino acids) and a liquid biological fertilizer (amino acids plus strain SQR9). Then, field experiments were performed to explore the crop yield enhancement efficiency using cowpea (*Vigna unguiculata*) as a model plant. This system was also selected as a model to investigate to what extent and how specifically leaf microbiota can be manipulated through inputs. Overall, the aims of this study were to (1) analyze the crop yield efficiency by foliar spray containing different inputs; (2) explore leaf microbiota variation after application of different liquid fertilizers; and (3) decipher the indicator for particular cropping practices (liquid fertilizer vs. liquid biological fertilizer) contributing to high crop yield.

## Methods

### Ethics statement

Our study was carried out on the farmers’ land (31°43'N, 118°46'E) at the Nanjing Institute of Vegetable Science, Nanjing, China and the leader of the institute Zhongyang Huang should be contacted for future permissions. No specific permits were required for the described field studies and the locations are not protected. The field studied did not involve endangered or protected species.

### Field description

Two seasons of continuous field experiments were performed at the Nanjing Institute of Vegetable Science, Nanjing, China (31°43'N, 118°46'E). This region has a tropical monsoon climate with an average annual temperature and precipitation of 15.4 °C and 1106 mm, respectively. The field soil before the experiment establishment had a pH value of 6.7 and contained 21.3 g kg^-1^ organic matter, 1.43 g kg^-1^ total nitrogen, 185 mg kg^-1^ available phosphorus and 242 mg kg^-1^ available potassium.

A 2-season field experiment was performed from August 2015 to June 2016 and included the following three treatments: (1) CF treatment, leaves sprayed with chemical fertilizer; (2) AA treatment, leaves sprayed with amino acid liquid fertilizer; and (3) AA9 treatment, leaves sprayed with liquid biological fertilizer (amino acid liquid fertilizer mixed with *B*. *amyloliquefaciens* SQR9). Each treatment had three randomized independent replications. The amino acid liquid fertilizer was produced as follows: pig hairs from the slaughterhouse were washed and dried. After that, the pig hair was put in an acid hydrolysis reactor with 3–4 mol L^-1^ sulfuric acids to material ratio of 1:2 (weight/volume). After 5–6 hours acid hydrolysis in 105–110 °C, the amino acid solution which concentration was more than 100 g L^-1^ was obtained. Then, a certain proportion of trace elements such as Fe, Mn, Cu, Zn, B, and Mo were poured in the stirred tank with the amino acid solution. Finally, amino acid liquid fertilizer was obtained after all the trace elements were dissolved. The amino acid liquid fertilizer contained total amino acids higher than 100 g kg^-1^, total N, total P, and total K contents of 29.7 g kg^-1^, 2.9 g kg^-1^ and 18.8 g kg^-1^, respectively, and the liquid biological fertilizer was amended with 1% of liquid fermented strain SQR9 cells (concentrations higher than 10^9^ CFU mL^-1^) to produce the new formulation. All treatments were amended with 6000 kg ha^-1^ of organic fertilizer and 750 kg ha^-1^ of compound chemical fertilizer (N+P_2_O5+K_2_O≥45%) as basal fertilizers. The organic fertilizer was produced by Nantong Huinong Co. Ltd, Jiangsu, China, by composting chicken manure at 30–70 °C for more than 20 days. All liquid fertilizers were adjusted to the same amount of N (29.7 g kg^-1^), P (2.9 g kg^-1^) and K (18.8 g kg^-1^) for each season using mineral fertilizers as necessary and surfactant was not added. In every season, the liquid fertilizers were sprayed four times at an interval of 1 week and started from the seedlings stage (beginning from August 19 in 2015 and April 2 in 2016). For each time, all liquid fertilizers were diluted 500 times by water and sprayed on plant leaves twice in the afternoon.

### Cowpea yield assay

For the total cowpea yield of each plot, all mature cowpea fruits were harvested and weighed. The fruit yield from each crop season (1^st^: autumn; 2^nd^: spring) was analyzed in this study. The agronomic characteristics (plant height and stem diameter) were measured after transferring the seedlings for 22 days.

### Leaf sampling, DNA extraction and Illumina MiSeq sequencing

Leaf sampling was performed in Jun. 2016, one day after the last spray during cowpea harvesting. Soon after, 6 plants in each pot were randomly selected, 9 leaves from one randomly selected plant were collected, and 54 leaves were mixed as a subsample for each treatment. Thus, 3 subsamples were collected for each treatment. The leaves were macerated by a liquid nitrogen grinding method, and DNA extraction was performed using the PowerPlant^®^ Pro DNA Isolation Kit (MoBio Laboratories Inc., Carlsbad, USA) according to the manufacturer’s protocol. The quality and concentration of the DNA samples were determined using a spectrophotometer (NanoDrop 2000, USA).

The DNA of each leaf sample served as a template for the amplification of the 16S rRNA gene and the ITS1 region. The V5-V6 region of the bacterial 16S rRNA gene was amplified using primers 799F (5'-AACMGGATTAGATACCCKG-3') and 1115R (5'-AGGGTTGCGCTCGTTG-3'), and ITS1F (5'-CTTGGTCATTTAGAGGAAGTAA-3') and ITS2 (5'-GCTGCGTTCTTCATCGATGC-3') were used for the ITS1 region of the fungal ITS gene. The programs for amplification and sequencing of the 16S and ITS genes were performed at Personal Biotechnology Co., Ltd. (Shanghai, China) on the Illumina MiSeq instrument. All sequences were deposited in the NCBI Sequence Read Archive database with the accession number (SRP161560).

### Bioinformatics analysis

Quality control and annotation of the raw sequences were performed according to Liu et al. [24]. A total of 21,050 16S rRNA and 17,901 ITS gene sequences for each sample were randomly selected for further bacterial and fungal microbial community analysis, respectively. To compare the similarities and differences of the bacterial and fungal community compositions among all soil samples, nonmetric multidimensional scaling (NMDS) based on the Bray-Curtis distance metric was performed using MOTHUR software [24], and analysis of molecular variance (AMOVA) was performed to evaluate the significant differences in bacterial and fungal community structures among the three treatments. AMOVA was used to compare the relative abundance of different groups according to the ordination base on OTU. In addition, Pearson’s correlation coefficient was used to evaluate the correlation between treatments, microbial diversity and cowpea yield. To evaluate the contribution of amino acids and microbial agents to cowpea yield and the yield promotion mechanism, variance partitioning analysis (VPA) and structural equation model (SEM) were carried out via the vegan and lavaan packages of R (version 3.3.1).

### Statistical analysis

The differences among the different treatments were assessed using a one-way ANOVA analysis, and the calculated means were subjected to Duncan’s multiple range test at *P* < 0.05. All analyses were performed in SPSS v18.0 (SPSS Inc., USA).

## Results

### Effects of different fertilization management programs on cowpea yield

**Fig 1:**
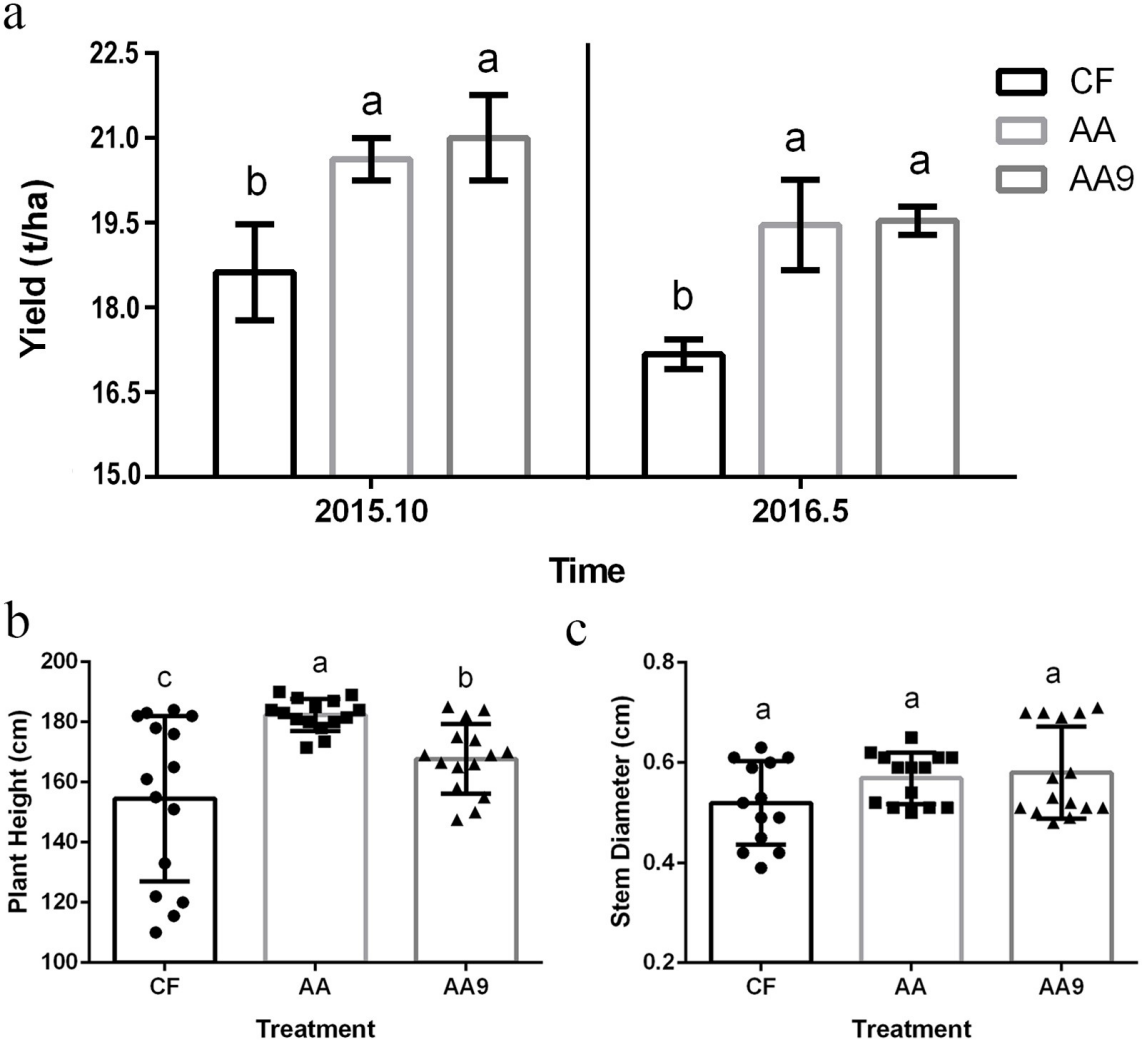
Effects of spraying different fertilizers on cowpea biomass. Effects of spraying different fertilizers on cowpea yields (a; Mean ± SD, n = 3) and plant height (b; Mean ± SD, n = 15) over two seasons, and stem diameter (c; Mean ± SD, n = 15) in the first season. CF, treatment sprayed with chemical fertilizer; AA, treatment sprayed with amino acids liquid fertilizer; AA9, treatment sprayed with liquid biological fertilizer. Different letters in the same line indicate significant differences as defined by Duncan’s test (*P* < 0.05).

As shown in Fig 1, cowpea yields in treatments sprayed with amino acid liquid fertilizer (AA) and liquid biological fertilizer (AA9) were significantly higher than those sprayed with CF in all crop seasons (Fig 1a). For the two seasons, the application of amino acid liquid fertilizer (AA) and liquid biological fertilizer (AA9) significantly (*P* < 0.05) increased the yield by 10.7% and 12.7%, respectively, compared to the CF treatment. These results indicated that the fertilization treatments (AA and AA9) persistently increased cowpea crop yields compared to the CF treatment. Moreover, in the first season, spraying amino acid liquid fertilizer (AA) significantly improved plant height compared to treatments with liquid biological fertilizer (AA9) and chemical fertilizer (CF), and significant enhancement was also observed in AA9 compared to CF (Fig 1b). For stem diameter, plants treated with AA and AA9 showed higher values than those treated with CF but there was no significant difference (Fig 1c)).

### Sequencing results

After basal quality control, a total of 219,848 16S rRNA and 217,508 ITS sequences were obtained for all soil samples. The number of high-quality sequences per sample varied from 21,050 to 27,853 for bacteria and from 17,901 to 30,385 for fungi. Moreover, at the 97% similarity cut-off level, 315 bacterial and 582 fungal OTUs were obtained.

### Shifts in microbial community richness and diversity

**Fig 2:**
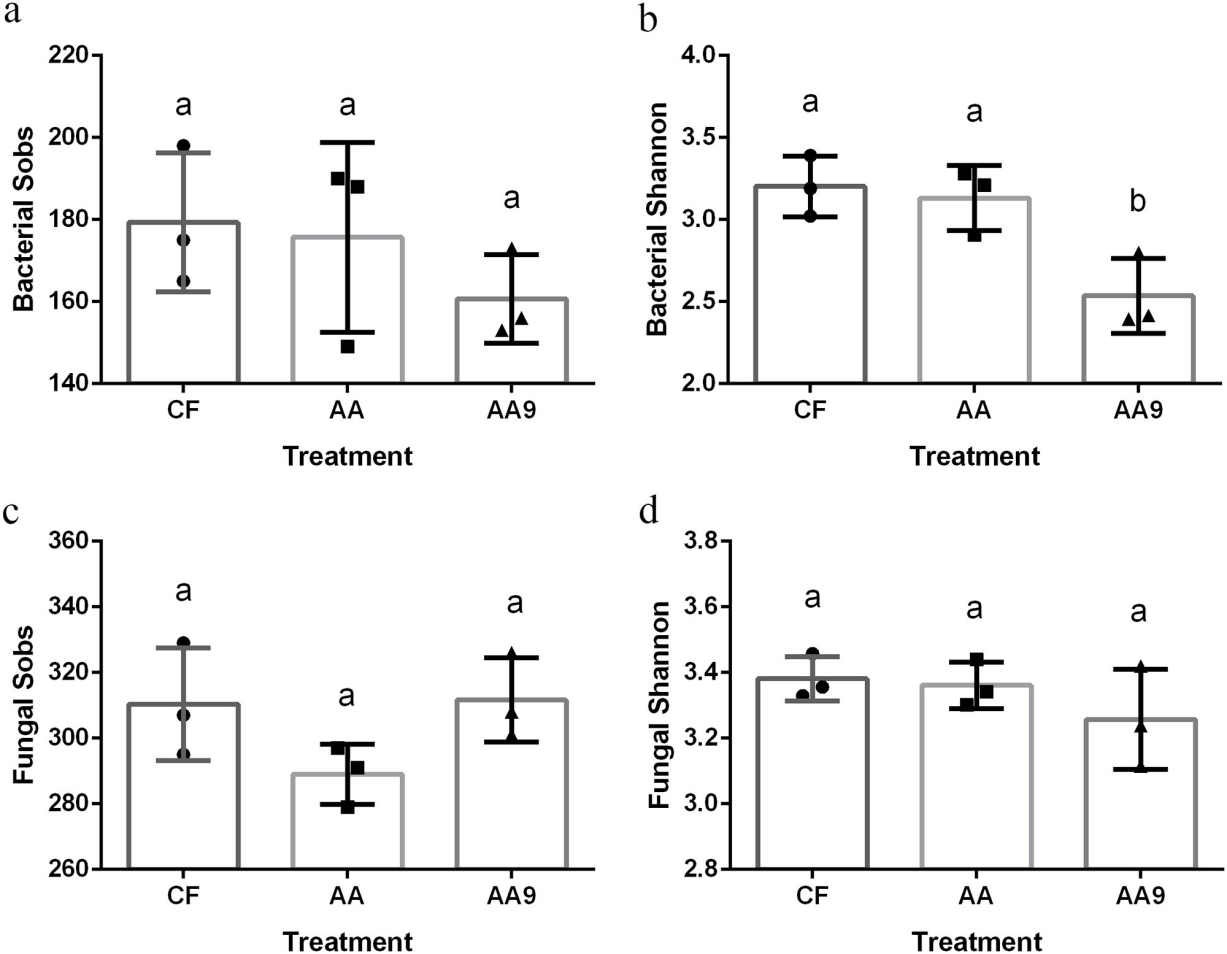
Bacterial and fungal α diversity. Bacterial and fungal richness (Sobs) and diversity (Shannon) indices in different treatments corresponding to different fertilization treatments. CF, treatment sprayed with chemical fertilizer; AA, treatment sprayed with amino acids liquid fertilizer; AA9, treatment sprayed with liquid biological fertilizer. Different letters in the same line indicate significant differences as defined by Duncan’s test (*P* < 0.05).

Bacterial and fungal observed richness (Sobs) and diversity (Shannon) indices were calculated based on the rarefied sequences (Fig 2). No significant difference was observed for Sobs, regardless of bacteria and fungi composition (Fig 2a and 2c). Furthermore, a significantly lower diversity (Shannon) of bacteria was noted for the treatment sprayed with liquid biological fertilizer (AA9) with the letter b in the above of the column (*P* < 0.05) (Fig 2b), while for fungi, no significant difference was observed (Fig 2d).

### Shifts in microbial community composition

**Fig 3:**
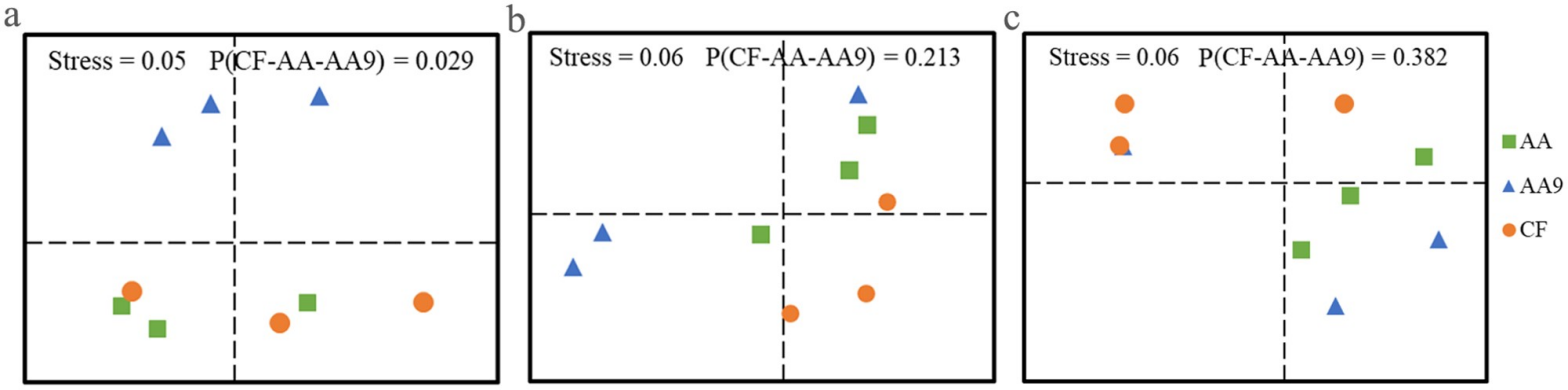
Bacterial and fungal composition. NMDS result showed the bacterial (a, with *Bacillus*; c, without *Bacillus*) and fungal (b) microbial community compositions of the different treatments. CF, treatment sprayed with chemical fertilizer; AA, treatment sprayed with amino acids liquid fertilizer; AA9, treatment sprayed with liquid biological fertilizer. The P value was calculated through AMOVA.

NMDS and AMOVA analyses indicated that bacterial (*P* = 0.029) (Fig 3a) community composition significantly differed but the result of fungi showed none significant difference (*P* = 0.213) (Fig 3b). The bacterial community structures in the AA9 treatment differed from those in the AA and CF treatments. Interestingly, after removing the OTUs belonging to *Bacillus*, no significant differences in bacterial community structures were also observed (*P* = 0.382) for bacteria, suggesting that the inoculation of functional microbes resulted in the difference (Fig 3c).

### Driving factor connected to yield enhancement

**Fig 4:**
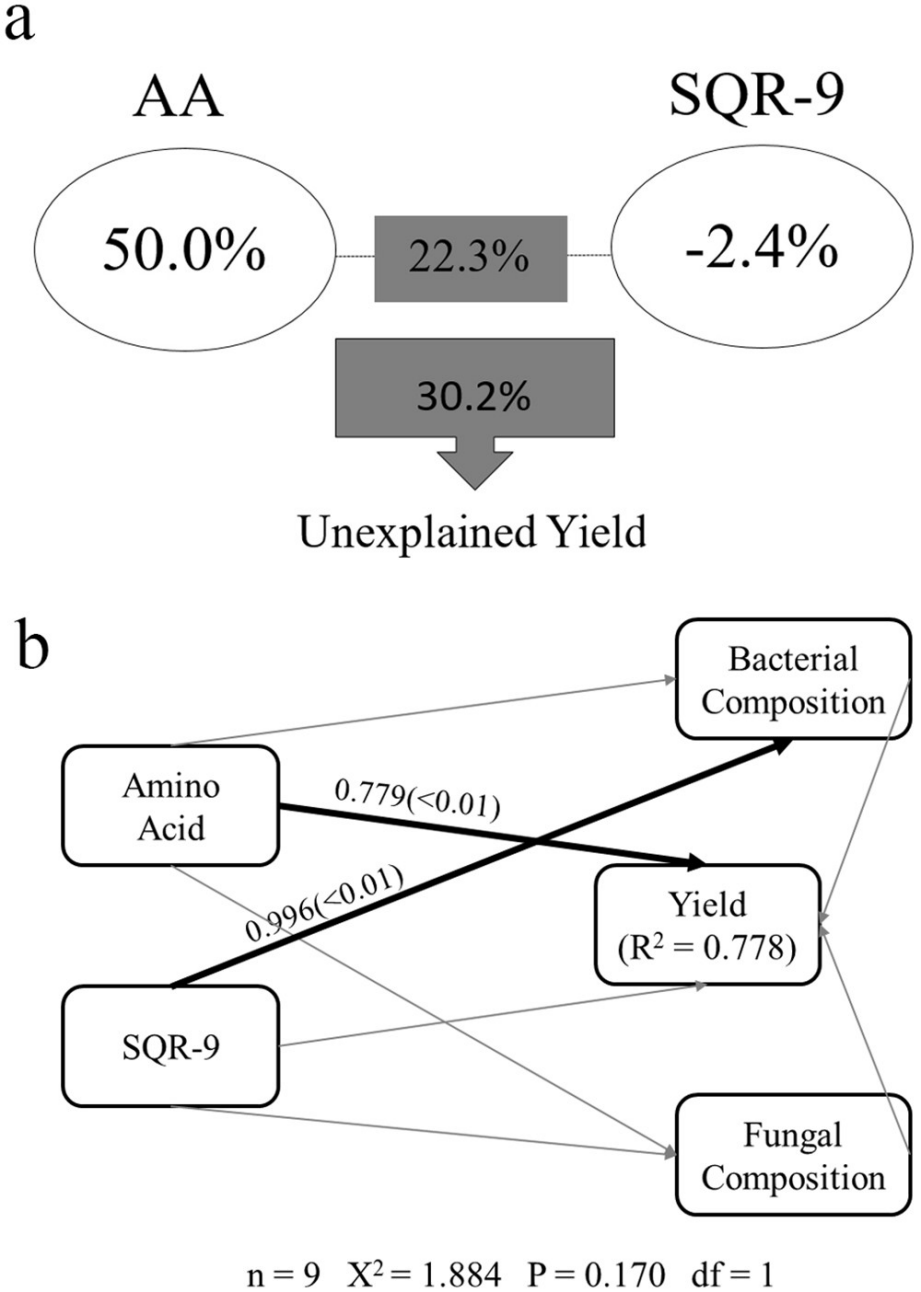
Mechanism of crop yield promotion. Variance partitioning analysis (VPA) map of the effects of amino acids, SQR9 and their interactions on the crop yields (a) and Structural Equation Modeling (SEM) analysis of a hypothesized network of linkages among amino acids, SQR9, bacterial composition, fungal composition and crop yield. AA, amino acids fertilizer; SQR9, functional PGPR strain SQR9 (b). The index was constructed by 0 when the factor was inexistence and 1 when the factor was positive.

As shown by the Pearson correlation analysis, amino acid liquid fertilizer significantly correlated with the crop yield (r = 0.868, *p* = 0.002), while functional strain SQR9 (r = 0.550, *p* = 0.125), bacterial Sobs (r = -0.523, p = 0.149) and Shannon (r = -0.642, *p* = 0.062) and fungal Sob (r = -0.122, p = 0.755) and Shannon (r = -0.267, *p* = 0.488) showed no significant relationship (Table 1). Moreover, amino acid liquid fertilizer, functional strain SQR9, and their interaction explained 50.0%, -2.4% and 22.3%, respectively, of the observed variation, leaving 30.2% of the variation unexplained for yield enhancement, as revealed by VPA analysis (Fig 4a).

**Table 1:** Pearson correlation analysis between different indicators and crop yields.

	AA	SQR-9	Bacteria		Fungi	
			Sobs	Shannon	Sobs	Shannon
r	0.868	0.550	-0.523	-0.642	-0.122	-0.267
p	0.002	0.125	0.149	0.062	0.755	0.488

Note: AA, amino acids fertilizer; SQR9, functional PGPR strain SQR9. The index was constructed by 0 when the factor was inexistence and 1 when the factor was positive.

Our multivariate causal model linking amino acid liquid fertilizer, functional strain SQR9, bacterial composition, fungal composition and yield was supported by the data (χ^2^ = 1.884, df = 1, *P* = 0.170; Fig 4). The exploratory SEM explained 77.8% of the variation in the yield. As shown in the model (Fig 4), consistent with the Pearson correlation analysis and VPA analysis, amino acid liquid fertilizer played a crucial role in yield enhancement, while functional strain SQR9 drove the bacterial composition.

### Microbial composition variation induced by liquid biological fertilizer

**Fig 5:**
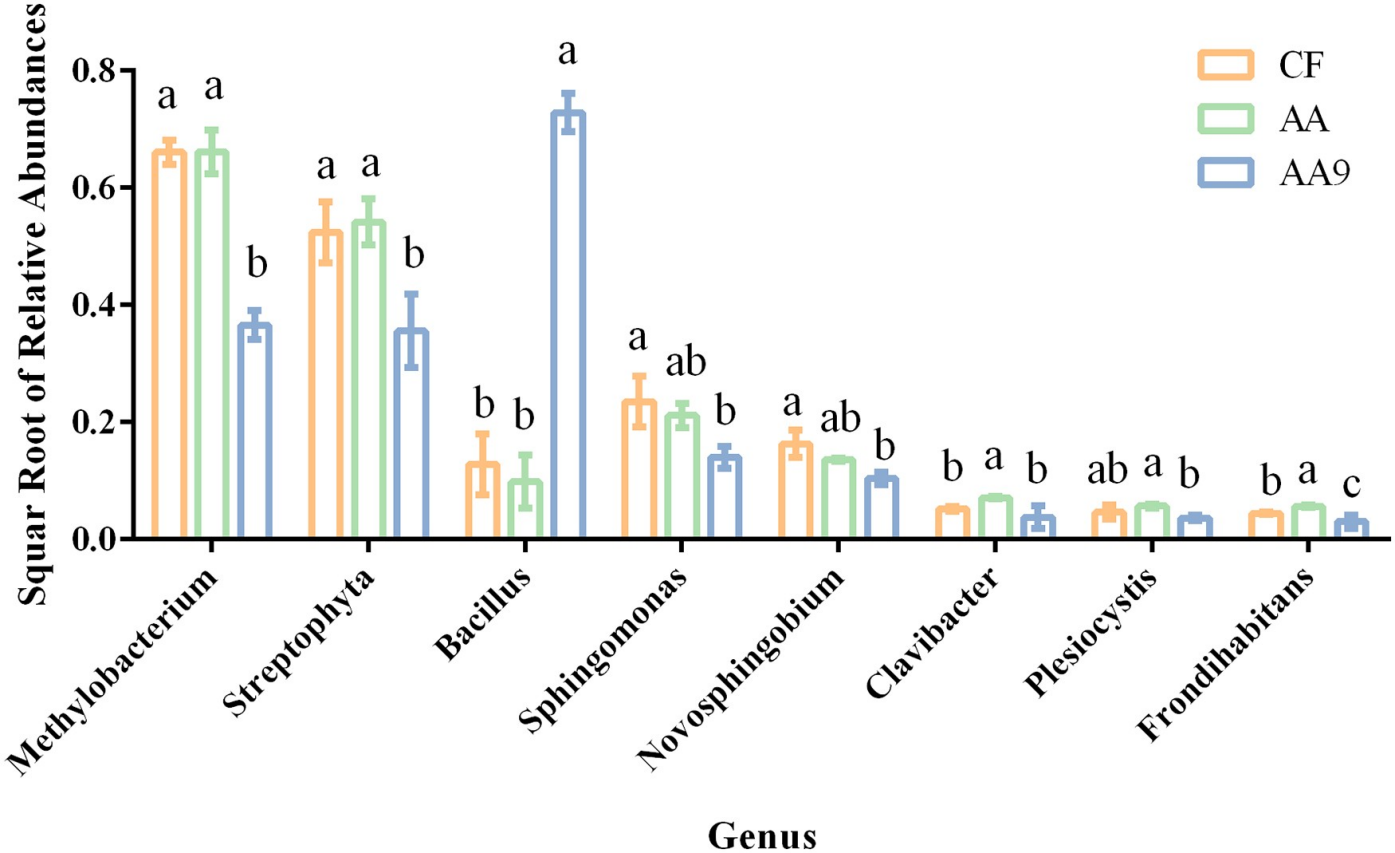
Microbial composition variation. Square root of the relative abundances (Mean ± SD, n = 3) of different genera with significant differences in different treatments. CF, treatment sprayed with chemical fertilizer; AA, treatment sprayed with amino acids liquid fertilizer; AA9, treatment sprayed with liquid biological fertilizer. Different letters in the same line indicate significant differences as defined by Duncan’s test (*P* < 0.05).

At the genus level (Fig 5), the abundance of *Bacillus* in treatments with liquid biological fertilizer (AA9) was significantly higher than that in treatments applied with amino acid liquid fertilizer (AA) and chemical fertilizer (CF). In contrast, the abundances of *Methylobacterium*, *Frondihabitans*, and *Streptophyta* were significantly lower in AA9 than in other treatments. Moreover, the values of *Clavibacter* and *Plesiocystis* were significantly lower in AA9 than in AA.

## Discussion

In our previous study, we observed that compared to non-treated plants (CK1) and plants treated with equal volume water (CK2), spray of amino acid liquid fertilizer (AA) significantly increased Cowpea yield [8]. Thus, the chemical fertilizer treatment as control to test the effects of foliar application of an amino acid liquid fertilizer, with or without PGPR strain SQR9, on the yield enhancement of cowpea in a two-season field experiment. Compared to chemical fertilizer, a significant yield enhancement effect was observed with the spraying of amino acid liquid fertilizer and liquid biological fertilizer treatments. These results were in agreement with the reports of other researchers who evaluated the effects of amino acids on the yield and/or growth of common bean, two wheat cultivars and *Urtica pilulifera* plants and suggested that foliar application of amino acid liquid fertilizer showed pleasant results [6, 10, 11]. However, these previously reported amino acid liquid fertilizers were produced by one or a solution composed of different amino acids, whereas the amino acids used in the present study were created from animal hairs resulted from the slaughterhouse. Due to the improvement of Chinese living standards, the increasing development of animal husbandry was induced [24, 29], especially for the scale and consumption of pork causing a huge amount of animal hair waste in the slaughterhouse that can generate a great risk to the environment. Thus, this study provides an effective and ecological leaf fertilization method based on amino acids created from dead animals that will not only enhance crop yield but also make full use of animal hairs to protect the environment. In addition, additional yield enhancement has also been achieved, and the results were in agreement with previous reports that PGPR strain SQR9 promoted crop growth [13, 14]. However, no significant difference was observed between foliar application of amino acid liquid fertilizer with or without PGPR strain SQR9, which may be due to the masking effect induced by amino acids, which showed impressive yield enhancement. Moreover, application of strain SQR9 showed significant lower plant height compared to none application, this may be due to that part of the nutrients in the liquid biological fertilizer is used by the bacteria and nutrient competition between plants and microbes have already been reported [30].

No significant differences in bacterial and fungal richness (Sobs), diversity (Shannon) and composition (NMDS) were identified between spraying amino acid liquid fertilizer and chemical fertilizer. Richness (Sobs), diversity (Shannon) and composition were the three key factors to describe the general microbiota characteristics [31], and the function of microbiome in the leaf surface have already been reported [32]; however, few reports to our knowledge focused on the leaf microbiota alteration via amino acid fertilization. Therefore, our findings here suggest that amino acid liquid fertilizer application induced less variation of leaf microbial diversity and composition. In the liquid biological fertilizer treatment application, significantly lower bacterial diversity (Shannon) and differences in bacterial composition (NMDS) were observed than in the other treatments, which may be due to the amendment of functional bacterial cells, which disturbed the leaf microbial community through colonization and was also supported by the NMDS analysis based on the data when *Bacillus* was removed. A similar phenomenon was observed in different environments in which one microbe invasion affected the indigenous microbiome [24, 33], suggesting that inoculation of PGPR strain *Bacillus amyloliquefaciens* SQR9 can also alter leaf microbial composition which can also be supported by the genus level results (Fig 5).

Pearson correlation analysis, VPA analysis, and SEM all showed that amino acid liquid fertilizer application, but not the functional strain and altered microbiota, was direct driver of yield enhancement. This result is supported by previous reports that showed the efficiency of amino acid uptake by plants [34, 35]. The results are also in accordance with the results of yield enhancement in this study and in previous studies [6, 10, 11] and with the general finding that amino acid liquid fertilizer treatments showed no significant effect on microbial composition and general microbiota characteristics. Moreover, in accordance with the results from NMDS and genera-level analysis, a significantly higher abundance of *Bacillus* was observed in the treatment sprayed with liquid biological fertilizer (AA9) than in the other two treatments, suggesting that *Bacillus amyloliquefaciens* SQR9 drove the bacterial composition. We have done the further analysis using taxonomic tree to do the key OTUs identification, and the OTUs belonging to *Bacillus* were selected. However, after using the Illumina sequencing primer pairs to search the matched segment from the whole genome of strain SQR9 which was download from Genebank, we found the matched segment wasn’t located in the 16S rRNA but in another place; thus the matched segment and 16s rRNA of SQR9 were included in the tree together (S1 Fig, the tree was built by maximum likelihood method including 10 more 16S rRNA sequences of type *Bacillus* species). From the tree, we found that one OUT (OTU4) was most similar to SQR9, and then we deduced that the functional PGPR strain SQR9 could efficiently colonize the leaf and alter the leaf microbial community. Although we did not identify the disease suppression ability and find significantly additional yield enhancement in this study, the colonization of functional microbes was still speculated to have positive functions on crop productivity. This is due to that in addition to some studies which showed disease suppression abilities of different bacteria isolated from rhizosphere when they were sprayed on the leaves [21, 36, 37] and observed varied effectiveness in causing localized disease inhibition when applied to leaves [38], plant growth-promoting rhizobacteria (PGPR) sprayed on plant leaves were also found to directly promote plant growth [22, 23]. However, this issue should be investigated in the future to test the direct plant growth promotion by avoid the masking effect from other nutrients.

## Conclusion

In the present study, leaves sprayed with amino acid liquid fertilizer (AA) and liquid biological fertilizer (AA9) experienced significantly enhanced cowpea yields compared to treatments with chemical fertilizer. Functional PGPR strain SQR9 was observed to efficiently colonize the leaf and alter the leaf microbial community, while amino acid liquid fertilizer treatment did not significantly alter the leaf microbiota. Of greatest interest is that amino acid liquid fertilizer resulted in the great degree of yield enhancement. Moreover, we speculate that foliar sprayed PGPR may have positive function due to the colonization of the beneficial microbe; however, more work should be done to make the effect of PGPR clear.

## Supporting information

S1 Fig Classification of OTU4. Taxonomic tree of OTU4 built by maximum likelihood method including 10 more 16S rRNA sequences (GQ360077.1) of type *Bacillus* species and matched segment (CP0068901.1) from the whole genome of strain SQR9. a, taxonomic tree; b, matched segment from the whole genome and 16S rRNA sequence of strain SQR9.
(DOCX)
