# Supplementary material for: Effects of foliar application of amino acid liquid fertilizers, with or without Bacillus amyloliquefaciens SQR9, on cowpea yield and leaf microbiota
Source: PLoS One. 2019 Sep 4;14(9):e0222048. doi: 10.1371/journal.pone.0222048 (PMC6726186; doi:10.1371/journal.pone.0222048)
Supplement: S1 Fig — Taxonomic tree of OTU4 built by maximum likelihood method including 10 more 16S rRNA sequences (GQ360077.1) of type Bacillus species and matched segment (CP0068901.1) from the whole genome of strain SQR9. a, taxonomic tree; b, matched segment from the whole genome and 16S rRNA sequence of strain SQR9. (DOCX) [file pone.0222048.s001.docx]

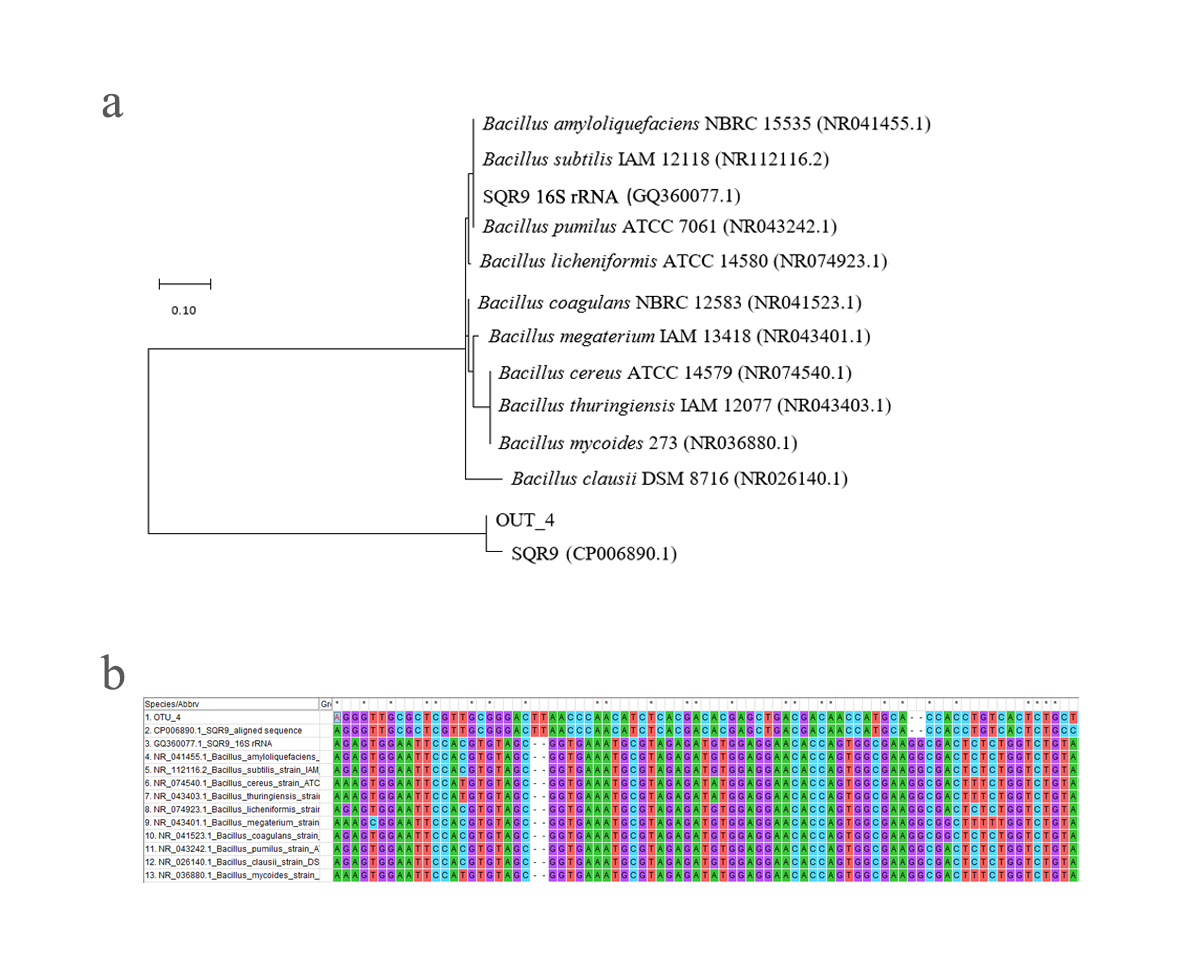


**S1 Fig Classification of OTU4.** Taxonomic tree of OTU4 built by maximum likelihood method including 10 more 16S rRNA sequences (GQ360077.1) of type *Bacillus* species and matched segment (CP0068901.1) from the whole genome of strain SQR9. a, taxonomic tree; b, matched segment from the whole genome and 16S rRNA sequence of strain SQR9.
